# Environmental and cortisol-mediated control of Ca^2+^ uptake in tilapia (*Oreochromis mossambicus*)

**DOI:** 10.1007/s00360-016-0963-7

**Published:** 2016-02-08

**Authors:** Chia-Hao Lin, Wei-Chun Kuan, Bo-Kai Liao, Ang-Ni Deng, Deng-Yu Tseng, Pung-Pung Hwang

**Affiliations:** Institute of Cellular and Organismic Biology, Academia Sinica, Taipei, Taiwan, ROC; National Institute for Basic Biology, National Institutes of Natural Sciences, Okazaki, Aichi Japan; Department of Biological Sciences and Technology, National University of Tainan, Tainan, 70005 Taiwan, ROC

**Keywords:** ECaC, Ca^2+^ influx, Ionocyte, Cortisol, Tilapia

## Abstract

Ca^2+^ is a vital element for many physiological processes in vertebrates, including teleosts, which live in aquatic environments and acquire Ca^2+^ from their surroundings. Ionocytes within the adult gills or larval skin are critical sites for transcellular Ca^2+^ uptake in teleosts. The ionocytes of zebrafish were found to contain transcellular Ca^2+^ transporters, epithelial Ca^2+^ channel (ECaC), plasma membrane Ca^2+^-ATPase 2 (PMCA2), and Na^+^/Ca^2+^ exchanger 1b (NCX1b), providing information about the molecular mechanism of transcellular Ca^2+^ transports mediated by ionocytes in fish. However, more evidence is required to establish whether or not a similar mechanism of transcellular Ca^2+^ transport also exists in others teleosts. In the present study, *ecac*, *pmca2*, and *ncx1* were found to be expressed in the branchial ionocytes of tilapia, thereby providing further support for the mechanism of transcellular Ca^2+^ transport through ionocytes previously proposed for zebrafish. In addition, we also reveal that low Ca^2+^ water treatment of tilapia stimulates Ca^2+^ uptake and expression of *ecac* and *cyp11b* (the latter encodes a cortisol-synthesis enzyme). Treatment of tilapia with exogenous cortisol (20 mg/l) enhanced both Ca^2+^ influx and *ecac* expression. Therefore, increased *cyp11b* expression is suggested to enhance Ca^2+^ uptake capacity in tilapia exposed to low Ca^2+^ water. Furthermore, the application of cortisol receptor antagonists revealed that cortisol may regulate Ca^2+^ uptake through glucocorticoid and/or mineralocorticoid receptor (GR and/or MR) in tilapia. Taken together, the data suggest that cortisol may activate GR and/or MR to execute its hypercalcemic action by stimulating *ecac* expression in tilapia.

## Introduction

The maintenance of Ca^2+^ homeostasis is important because Ca^2+^ is involved in many physiological activities, such as muscle contraction, neuron excitation, and bone formation in vertebrates (WendelaarBonga and Pang 1991). Fish, which live in aquatic environments with inconsistent Ca^2+^ levels, have to maintain their body fluid Ca^2+^ homeostasis through an efficient Ca^2+^ regulation mechanism. The major organ for ionoregulation in fish is the gills, which are responsible for over 95 % of Ca^2+^ uptake from water in freshwater-adapted species (Flik et al. 1995). The skin serves as the main organ for ionoregulation at early developmental stages of fish, before the gills are fully developed (Hwang et al. 1994, 2011). Ionocytes in the gills or larval skin are vital sites for ion uptake in fish (Hwang et al. 2011). In an early study in trout, branchial Ca^2+^ uptake was demonstrated to be active and transcellular (Perry and Flik 1988). The understanding of the Ca^2+^ absorption mechanism in fish gills or skin progressed swiftly after the discovery of epithelial Ca^2+^ channel (ECaC) (Qiu and Hogstrand 2004; Pan et al. 2005; Shahsavarani and Perry 2006); expression of ECaC mRNA and/or protein expression was specifically identified in the gills and/or skin ionocytes of zebrafish, trout, and medaka (Pan et al. 2005; Shahsavarani and Perry 2006; Liao et al. 2007; Hsu et al. 2014). Furthermore, Liao et al. (2007) revealed that ECaC, plasma membrane Ca^2+^–ATPase 2 (PMCA2), and Na^+^–Ca^2+^ exchanger 1b (NCX1b) are co-expressed in the same group of ionocytes in zebrafish (Liao et al. 2007). Based on the above studies, the following model of transcellular epithelial Ca^2+^ transport in the gills/skin was provided: external Ca^2+^ is absorbed through apical ECaC, and the absorbed Ca^2+^ is then extruded into the plasma by basolateral PMCA and NCX (Hwang et al. 2011).

The Mozambique tilapia (*Oreochromis mossambicus*), a euryhaline teleost, is capable of surviving up to approximately 4-times the salt content of seawater (Stickney 1986); this organism was previously used to investigate the correlation between the morphology of gill ionocytes and declining environmental Ca^2+^ (Chang et al. 2001). The regulation of Ca^2+^ balance in developing larvae is dependent upon external Ca^2+^ levels. Upon acute exposure to low Ca^2+^, both Ca^2+^ influx and net uptake were increased in newly hatched larvae (Hwang et al. 1996; Chou et al. 2002). When small-bodied or growing female tilapia were transferred to a low-Ca^2+^ environment, significant upregulation of Ca^2+^ influx in the tilapia was observed (Flik et al. 1986; Chang et al. 2001). Moreover, orthologues of zebrafish ECaC, PMCA2, and NCX1b have also been identified in tilapia (Pan et al. 2005; Liao et al. 2007). Based on findings in zebrafish (Liao et al. 2007; Hwang and Chou 2013), it may be assumed that apical ECaC and basolateral PMCA2 and NCX1 in ionocytes are responsible for transcellular epithelial Ca^2+^ transport in tilapia. However, there are no published accounts of comprehensive studies of the role of these Ca^2+^ transporters (ECaC, PMCA2, and NCX1) in Ca^2+^ regulation, or molecular evidence of their expression in ionocytes, in any fish species other than zebrafish.

Previous studies indicated that plasma cortisol levels are upregulated in trout exposed to a low ambient Ca^2+^ level (Perry and Wood 1985; Flik and Perry 1989). Lin et al. (2011) revealed that low Ca^2+^ water treatment stimulated expression of *cyp11b* (encoding an enzyme involved in the final step of cortisol synthesis) in zebrafish. Due to the hypercalcemic action of cortisol (Perry and Wood 1985; Flik and Perry 1989; Shahsavarani and Perry 2006; Lin et al. 2011), these responses were suggested to assist the maintenance of body fluid Ca^2+^ homeostasis in low Ca^2+^ environments. However, few studies have further explored the hypercalcemic effect of cortisol on transcellular epithelial Ca^2+^ transporters in fish. Cortisol treatment was shown to stimulate *ecac* mRNA expression in the gills of trout (Shahsavarani and Perry 2006). Moreover, *ecac* expression was found to be enhanced by cortisol treatment, while expression of both *pmca2* and *ncx1b* was unaffected in zebrafish embryos, suggesting that ECaC is a regulatory target of cortisol (Lin et al. 2011). However, it is unclear whether ECaC is the main target of cortisol signaling in terms of transcellular Ca^2+^ transport in teleosts other than zebrafish and trout. Hormones exert their activity by binding specific receptor(s). In cell lines transfected with teleost glucocorticoid receptor (GR) or mineralocorticoid receptor (MR), cortisol treatment activated the transcription of a glucocorticoid response element (GRE)-element containing plasmid (Trapp and Holsboer 1996; Colombe et al. 2000; Bury et al. 2003; Greenwood et al. 2003; Sturm et al. 2005). In addition, cortisol treatment affected the mRNA expression of different ion transporters through GR and/or MR in Atlantic salmon (Kiilerich et al. 2007). GR and MR mRNA signals were detected in the branchial ionocytes in tilapia (Aruna et al. 2012). Thus, cortisol may exert its hypercalcemic function through GR and/or MR in fish. Cortisol acts via GR, but not MR, to stimulate Ca^2+^ uptake and *ecac* expression in zebrafish (Lin et al. 2011), but it is unclear whether this regulation also occurs in other teleosts.

The purpose of the present study is to enhance our comprehensive understanding of fish Ca^2+^ transport and cortisol control in terms of body fluid Ca^2+^ homeostasis. We initially hypothesized that (1) ECaC, PMCA2, and NCX1 are responsible for transcellular epithelial Ca^2+^ transport in tilapia, and (2) cortisol acts via GR and/or MR to regulate Ca^2+^ uptake by modulating expression of these Ca^2+^ transporters in tilapia. To test these hypotheses, we designed experiments to answer the following specific questions: (1) are *ecac*, *pmca2*, and/or *ncx1* expressed in ionocytes in tilapia? (2) Does the external Ca^2+^ level regulate *ecac*, *pmca2*, and *ncx1* expression in tilapia? (3) Does cortisol modulate Ca^2+^ uptake and the mRNA expression of *ecac*, *pmca2*, and *ncx1* in tilapia? And finally, (4) does cortisol regulate Ca^2+^ uptake through the GR and/or MR?

## Materials and methods

### Animals

Tilapia (*Oreochromis mossambicus*), 1–50 g in body weight, were taken from stocks at the Institute of Cellular and Organismic Biology, Academia Sinica, and kept in freshwater (local tap water; [Ca^2+^], 0.20 mM; [Mg^2+^], 0.16 mM; [Na^+^], 0.5 mM; [K^+^], 0.3 mM; [Cl^−^], 0.45 mM) at 27 °C under a 14 h:10 h light:dark photoperiod. Tilapia larvae were acquired as follows: fertilized eggs were collected from the mouths of female tilapia and incubated in aerated FW. Fertilized eggs that hatched at the same time were used in the experiments. All experiments were conducted on yolk-sac larvae, and no feeding occurred. The incubation water was changed daily to control water quality. For sampling, fish (adult and hatched embryos) were anesthetized with buffered MS-222 (Sigma-Aldrich, USA) and then dissected. Sampling was performed in accordance with the guidelines of the Academia Sinica Institutional Animal Care and Utilization Committee (Approval No.:RFiZOOHP2002086).

### Acclimation experiment

Artificial fresh waters with high (2 mM) and low (0.02 mM) Ca^2+^ levels were prepared with double-deionized water (model Milli-RO60; Millipore, Billerica, MA, USA) supplemented with adequate CaSO_4_·2H_2_O, MgSO_4_·7H_2_O, NaCl, K_2_HPO_4_, and KH_2_PO_4_. The Ca^2+^ concentrations of the high- and low-Ca^2+^ media were 2 and 0.02 mM, respectively, but all other ion concentrations in all media were the same as those in local tap water ([Na^+^], 0.5 mM; [Mg^2+^], 0.16 mM; and [K^+^], 0.3 mM). Variations in ion concentrations were maintained within 10 % of the predicted values by monitoring with an atomic absorption spectrophotometer (Hitachi Z-8000, Tokyo, Japan). For acclimation, hatched embryos and adults were incubated with high- and low-Ca^2+^ media for 3 days and 2 weeks, respectively. Fish were sampled for the assay at the end of the acclimation period.

### Cortisol and receptor antagonist incubation

Cortisol dosages were selected with reference to previous studies (Lin et al. 1999, 2011, 2015a, b; Cruz et al. 2013a). Cortisol (hydrocortisone, Sigma-Aldrich, USA) was prepared as a stock solution in dimethyl sulfoxide (DMSO) first and then the stock was diluted to the final working solution (0, 10, and 20 mg/l) in local tap water. Hatched tilapia embryos were treated with cortisol media for 3 days and then were sampled for subsequent analysis. Incubation media were refreshed every day to maintain consistent levels of cortisol. During incubation, neither significant mortality nor abnormal behavior was observed. Doses of GR and MR antagonists were selected with reference to a previous study (Kiilerich et al. 2007). In this study, 10 µg/ml of RU486 (GR antagonist, Sigma-Aldrich, USA) or Spironolactone (MR antagonist, Sigma-Aldrich, USA) were used, and the medium was changed every day. Although used dosages of cortisol and antagonists in the present study are higher than in some studies (Pippal et al. 2011; Kumai et al. 2012), they had been proofed to work in cultured gills and fish larvae in previous studies (Lin et al. 1999, 2011, 2015a, b; Kiilerich et al. 2007; Cruz et al. 2013a). In addition, these used dosages did not cause the damage to tilapia larvae.

### Preparation of total RNA

After anesthesia with 0.03 % MS222 (Sigma), appropriate amounts of tilapia tissues or embryos were collected. For the RNA extraction, the samples were homogenized in 1 ml Trizol reagent (Invitrogen, Carlsbad, CA, USA) and then referred to manufacturer’s protocol. Finally, the quantity and quality of total RNA were assessed based on the absorbance at 260 nm and the ratio of the absorbance at 260 and 280 nm, as measured using a Nanodrop ND-2000 (Thermo Scientific, Wilmington, DE, USA).

### Reverse transcription-PCR analysis

The mRNA was purified from the total RNA extracted from tilapia tissues with a commercial kit (Oligotex, Qiagen, Hilden, Germany). For cDNA synthesis, 0.36 μg of mRNA was reverse transcribed in a final volume of 20 μl containing 0.5 mM dNTPs, 2.5 μM oligo (dT)_18_, 5 mM dithiothreitol, and 200 units PowerScript reverse transcriptase (Clontech, CA, USA) for 1.5 h at 42 °C, followed by a 15 min incubation at 70 °C. For PCR amplification, 2 μl cDNA was used as template in a 50 μl final reaction volume containing 0.25 mM dNTP, 2.5 units EX-Taq polymerase (Takara, Shiga, Japan), and 0.2 μM of each primer. GenBank accession numbers of the sequences for primer sets were used as follows: *ecac*, GenBank BankIt Submission ID:1884659; *pmca2*, AAK15034; *ncx1*, AY283779; *gadph*, FN673690.

The primer sets used for Reverse transcription-PCR analysis were as follows: *ecac* (342 bp fragment), forward 5′-AGAGGATGAAAAGGAAACGG-3′, reverse 5′-ATGGCATAATACTGCGGAAA-3′; *ncx1* (310 bp fragment), forward 5′-TGCCGTCTACCACTACACCC-3′, reverse 5′-GCAGCGACCTAAAATCCAAC-3′; *pmca2* (693 bp fragment), forward 5′-AACAACCTGGTGCGTCA-3′, reverse 5′-GGGGTCCTCTATTCCGA-3′; *gadph* (415 bp fragment), forward 5′-AATACGACCCCTCCTCCAT-3′, reverse 5′-TACCCCAGCACTCCTTTCA-3′. The amplicons were all sequenced to ensure that the PCR products were the desired gene fragments.

### In situ hybridization

PCR fragments of tilapia *ecac*, *pmca2*, and *ncx1* were obtained by PCR and inserted into a pGEM-T easy vector (Promega, WI, USA). After linearization by restriction enzyme digestion, the plasmids were subjected to in vitro transcription with T7 and SP6 RNA polymerase (Roche, Penzberg, Germany) to produce sense and anti-sense transcripts, respectively. Dig-labeled RNA probes were examined with RNA gels and dot-blot assay to confirm the quality and concentration.

Excised gills were fixed with 4 % paraformaldehyde for 3 h at 4 °C and then washed several times with phosphate buffered saline (PBS). Fixed samples were immersed in PBS containing 30 % sucrose overnight, and embedded in OCT compound embedding medium (Sakura, Tokyo, Japan) at −20 °C. Frozen cross-sections of 10 μm were cut with a CM 1900 rapid sectioning cryostat (Leica, Heidelberg, Germany) and attached to poly-l-lysine coated slides (Erie, New Hampshire, USA). After brief washing with PBST, slides were incubated with hybridization buffer (HyB) containing 50 % formamide, 5× SSC, and 0.1 % tween-20 for 5 min at 65 °C. Prehybridization was performed for 2 h at 65 °C with HyB^+^ (hybridization buffer with additional 500 ng/ml yeast tRNA and 50 μg/ml heparia). For hybridization, samples were incubated with100 ng RNA probe in 200 μl HyB^+^ at 65 °C overnight. Next, the slides were washed at 65 °C for 10 min in 75 % HyB and 25 % 2× saline sodium sitrate (SSC), 10 min in 50 % HyB and 50 % 2× SSC, 10 min in 25 % HyB and 75 % 2× SSC, 10 min in 2× SSC, and finally 30 min in 0.2× SSC at 70 °C (this final wash was repeated twice). Further washes were performed at room temperature for 5 min in 75 % 0.2× SSC and 25 % phosphate buffered saline with 0.1 % triton X-100 (PBST), 5 min in 50 % 0.2× SSC and 50 % PBST, 5 min in 25 % 0.2× SSC and 75 % PBST, and 5 min in PBST. After the series of washes, slides were incubated for 2 h in blocking solution containing 5 % sheep serum and 2 mg/ml bovine serum albumin (BSA) in PBST, and then incubated with 1:2500 antibody (Roche, Basel, Switzerland) in blocking solution for another 2 h at room temperature. Finally, sections were washed with PBST plus blocking reagent, and were then transferred to staining buffer. The staining reaction was performed with 5-bromo-4-chloro-3-indolyl phosphate (BCIP) and p-nitroblue tetrazolium chloride (NBT) in staining buffer until the signal was strong enough for analysis.

### Immunohistochemistry

Sections were washed several times with PBST after in situ hybridization. Blocking was performed in 3 % BSA at room temperature for 2 h, and sections were then incubated with α5 mouse anti-Na^+^/K^+^-ATPase (2.5 µg/ml in PBS) at 4 °C overnight. Samples were washed in PBS for 30 min twice, and then incubated with goat anti-mouse IgG conjugated with FITC (7.5 µg/ml in PBS; Jackson Immunoresearch Laboratories, West Grove, PA, USA) for 1 h at room temperature. Images were acquired with a Leica TCS-NT confocal laser scanning microscope (Leica Lasertechnik, Heidelberg, Germany).

### Quantitative-PCR

The mRNA purification and cDNA synthesis procedures are described above in the section for RT-PCR. Q-PCR reactions were performed with an ABI7000 sequence detection system (ABI, Warrington, UK) in a final volume of 20 μl containing 10 μl 2× SYBR Green master mix (ABI, Warrington, UK), 100 nM primer pairs, and 8 μl cDNA. The standard curve of each gene was checked in the linear range with GAPDH as an internal control. The primer sets used for Q-PCR were as follows: *cyp11b* (160 bp fragment), forward 5′-ACATCTTCAGTCATGCGGAG-3′, reverse 5′-ATGAGGTCCAAAGATAGCTGC-3′; *mr* (145 bp fragment), forward 5′-GCTGTGGAAGGTCAGCATAA-3′, reverse 5′-ACTTCTTGGATTTCCGTGCT-3′; *gr* (159 bp fragment), forward 5′-AAAGGCCAGCACAACTACCT-3′, reverse 5′-CTGGACACCCTTTAACCGAT-3′; *ecac* (152 bp fragment), forward 5′-CTGTCTCTGGCCTCGACTT-3′, reverse 5′- CCTCCGTTTCCTTTTCATCCT-3′; *pmca2*(157 bp fragment), forward 5′-TCTGTCAGGAAGTCGATGA-3′, reverse 5′-CCTTGTCTCGTGGACGGAA-3′; *ncx1*(261 bp fragment), forward 5′-CAAGAGAGCCACCCATGATATCTT-3′, reverse 5′-TTTCCGTCTCACCGGGTTT-3′; for *β*-*actin* (132 bp fragment), forward 5′-GGTGGGTATGGGTCAGAAAG-3′, reverse 5′-TGCCAGATCTTCTCCATGTC-3′’. The *β*-*actin* was used a house-keeping gene to normalize mRNA expression and its expression was not modulated in the experiments of Ca^2+^ and pharmacological treatment in this study.

### Measurement of Ca^2+^ influx

Measurement of Ca^2+^ influx was performed as described previously (Chen et al. 2003). High- and low-Ca^2+^ freshwater-acclimated tilapia were transferred to tracer media containing ^45^Ca^2+^. The plot of radioactivity against incubation time was linear within 8 h. Samples (200 μl) were collected from the tracer media at 0.5 and 2.5 h after transfer. Counting solution (Ultima Gold, Packard, USA) was added to the samples, and the radio activities were counted with a liquid scintillation β-counter (LS6500, Beckman, Fullerton, CA). Ca^2+^ influx rates were calculated using the following formula:

$$J_{\text{i}} = \, (Q_{\text{i}} \times \, V_{\text{i}} - \, Q_{\text{f}} \times V_{\text{f}} ) \, / \, \left( { 1/ 2 { } \times \, \left( {{\text{SA}}_{\text{i}} + {\text{ SA}}_{\text{f}} } \right) \, \times \, t \, \times \, W} \right) .$$

where *Q*_i_ and *Q*_f_ (cpm ml^−1^) refer to initial (0.5 h) and final (2.5 h) radioactivities in the tracer media, *V*_i_ and *V*_f_ (ml) refer to initial and final volumes of the tracer media, SA_i_ and SA_f_ are initial and final specific activities (cpm mmole^−1^), *t* (2 h) is incubation time, and *W* (*g*) is fish body weight.

### Statistical analysis

Group datasets were confirmed to be normally distributed by Anderson–Darling Normality test (*p* < 0.05). Data are presented as the mean ± SD and were analyzed by one-way analysis of variance (ANOVA) and Student’s *t* test.

## Results

### Expression of mRNA encoding Ca^2+^ transporters in different tissues

**Fig. 1:**
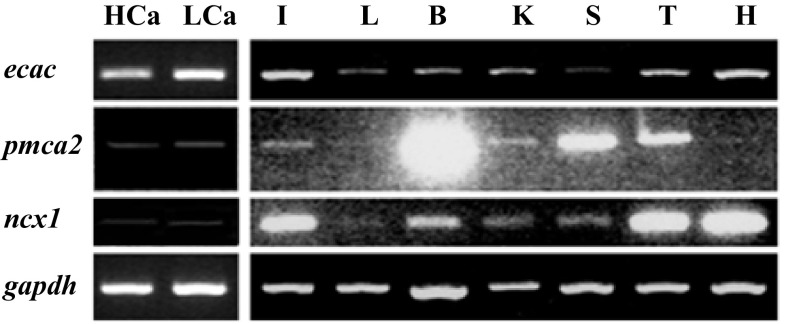
Expression of *ecac*, *pmca2*, and *ncx1* in various tissues of tilapia, as detected by RT-PCR. The *gadph* gene was used as internal control to evaluate the relative amounts of cDNAs. *I* intestine; *L* liver; *B* brain; *K* kidney; *S* spleen; *T* testis; *H* heart; *HCa* high-Ca^2+^ acclimated tilapia; *LCa* low-Ca^2+^ acclimated tilapia

The mRNA expression patterns of the tilapia genes encoding ECaC, PMCA2, and NCX1 were first evaluated by RT-PCR (Fig. 1). In adult fish, expression of *ecac*, *pmca2*, and *ncx1* were ubiquitous among all tissues examined, including brain, heart, gills, intestine, liver, spleen, testis, and kidneys.

### Localization of *ecac*, *pmca2*, and *ncx1b* in the gills

**Fig. 2:**
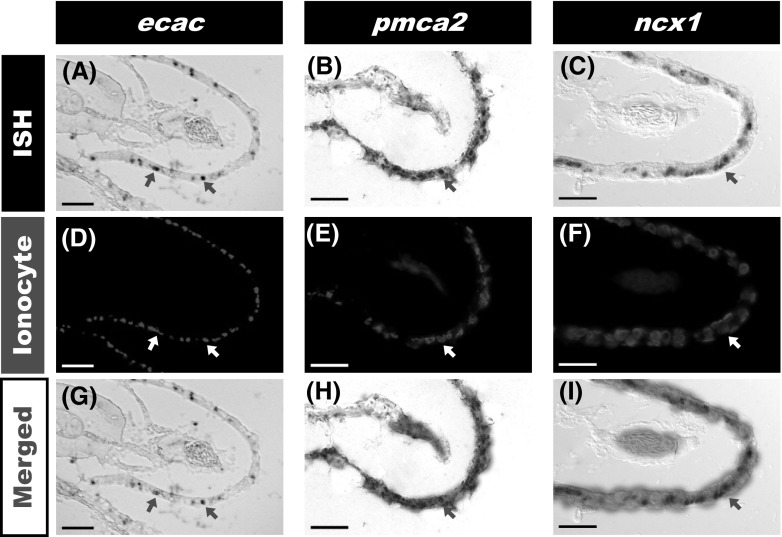
In situ hybridization against *ecac*, *pmca2*, and *ncx1* in tilapia gills. **a**–**c** Expression of *ecac, pmca2*, and *ncx1*; **d**-**f**, immunohistochemical staining of ionocytes using Na,K-ATPase *α*5 antibody; **g**-**i**, co-localization of ionocytes with *ecac*, *pmca2*,or *ncx1b* signals. *Arrows* indicate cells with positive signals. *Scale bar* 20 μm

Tilapia gills were removed and treated to prepare cryosections. Sections were subjected to in situ hybridization against tilapia ECaC, PMCA2,or NCX1 mRNA, and then double stained with Na^+^/K^+^ ATPase *α*5 (an ionocyte marker) antibody, revealing that *ecac*, *pmca2*, and *ncx1b* are expressed in the ionocytes of gill filaments (Fig. 2). All *ecac* signals were co-localized with ionocytes (Fig. 2g). However, only some *pmca2* and *ncx1* signals co-localized with ionocytes (Fig. 2h, i).

### Ca^2+^ influx and expression of branchial Ca^2+^ transporters in adult tilapia acclimated to low or high Ca^2+^ water

**Fig. 3:**
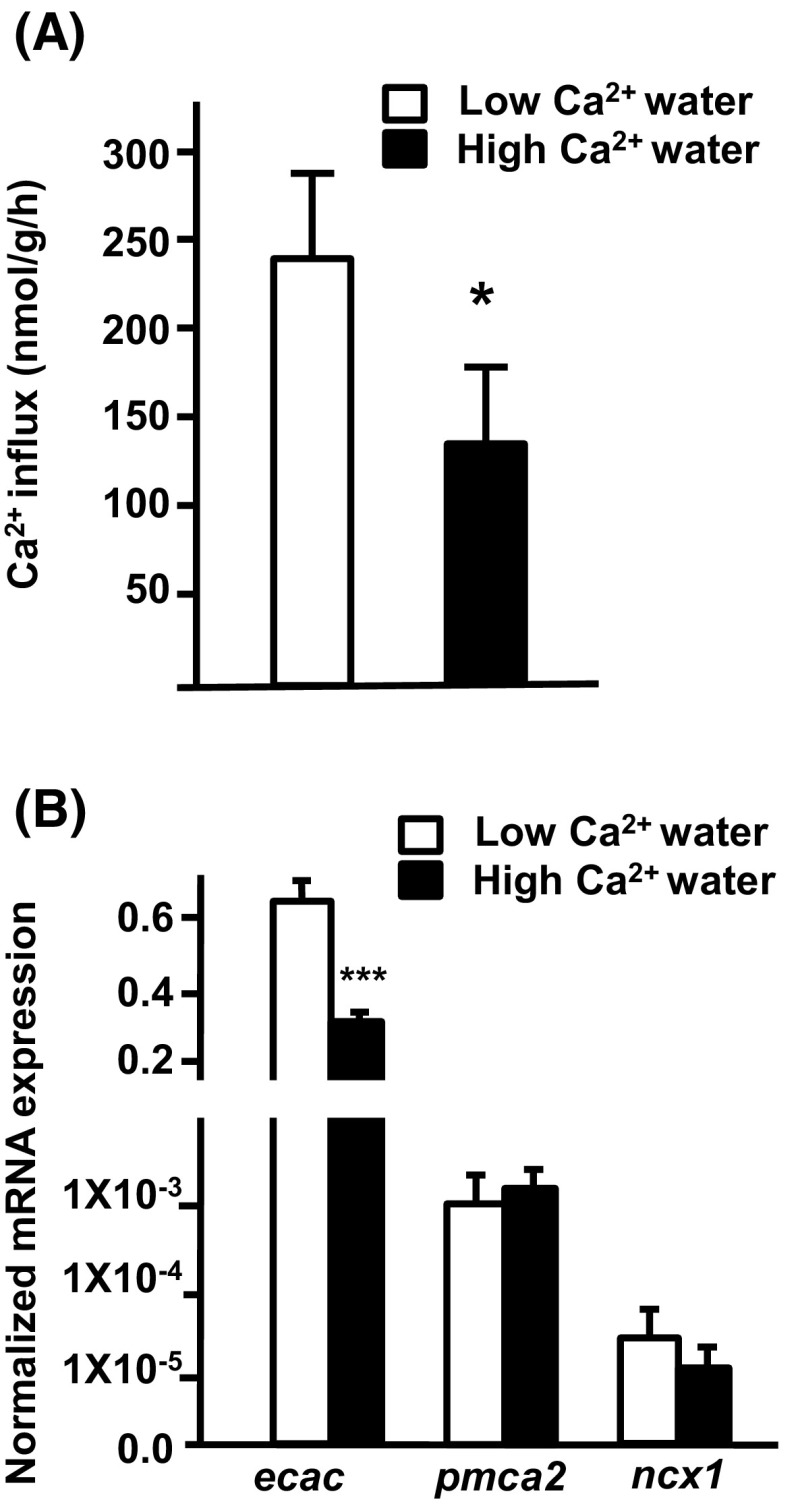
Effects of environmental Ca^2+^ on Ca^2+^ influx and mRNA expression of branchial transporters in tilapia. **a** Ca^2+^ influx. **b** Expression of mRNA, as analyzed by qPCR; values were normalized to β-actin. Values are the mean ± SD (*n* = 5–8). * *p* < 0.05; ***p < 0.001. Student’s *t* test

Tilapia were treated with low (0.02 mM) or high (2.0 mM) Ca^2+^ water for 2 weeks prior to sampling for investigation of Ca^2+^ influx and mRNA expression. Ca^2+^ influx of tilapia was higher in low than high Ca^2+^ water (Fig. 3a). Furthermore, branchial *ecac* expression was approximately threefold higher in tilapia acclimated to low Ca^2+^ than tilapia acclimated to high Ca^2+^ treatment. However, expression of branchial *pmca2* and *ncx1* was no different between treatments (Fig. 3b).

### Ca^2+^ influx and related gene expression in tilapia larvae treated with low or high Ca^2+^

**Fig. 4:**
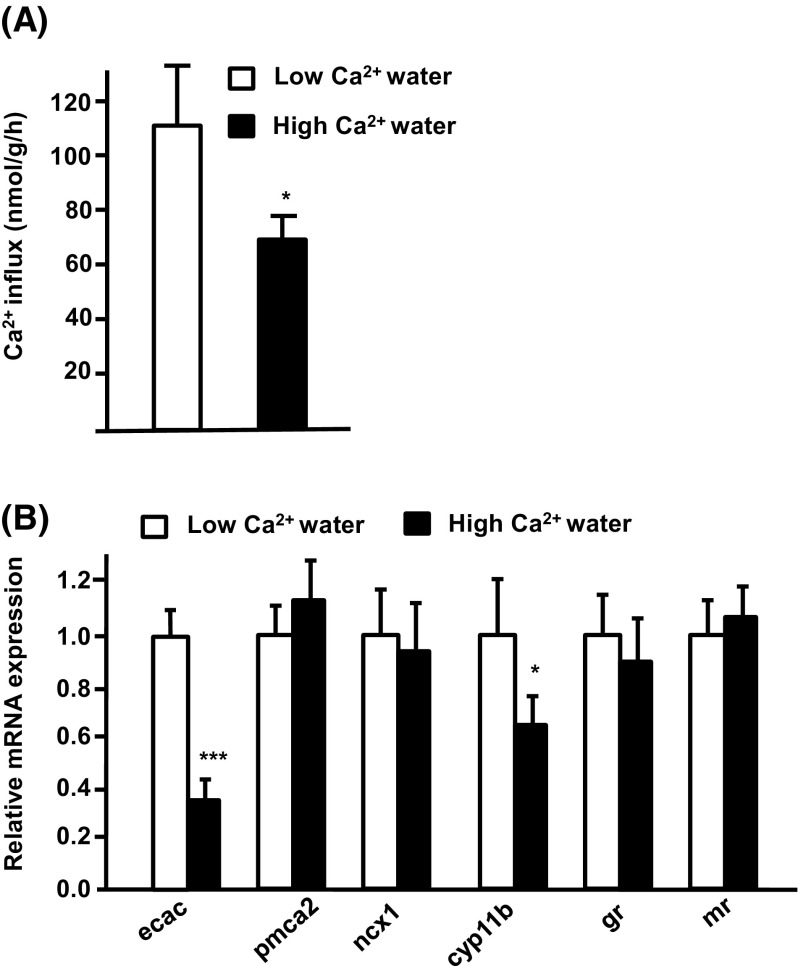
Effects of environmental Ca^2+^ on Ca^2+^ influx and mRNA expression of transporters in tilapia larvae. **a** Ca^2+^ influx; **b** expression of mRNA, as analyzed by qPCR; values were normalized to β-actin. Values are the mean ± SD (*n* = 6). * *p* < 0.05; ***p < 0.001. Student’s *t* test

The effects of different Ca^2+ ^levels on tilapia larvae were examined by acclimating hatching tilapia embryos to low (0.02 mM) or high (2.0 mM) Ca^2+^ water for 3 days. After 3 days, larvae were sampled for analysis of Ca^2+^ influx and gene expression. Similar to the adult, tilapia larvae acclimated to low Ca^2+^ water also exhibited significantly upregulated Ca^2+^ influx and *ecac* expression (Fig. 4a, b), while the expression of *pmca2* and *ncx1* was not modulated by external Ca^2+^ level (Fig. 4b). The role of cortisol in Ca^2+^ uptake in tilapia was further clarified by studying the expression of cortisol-related genes in tilapia larvae. The expression of *cyp11b* was significantly higher in larvae acclimated to low than to high Ca^2+^ medium, but the expression of *gr* and *mr* was not modulated (Fig. 4b).

### The effect of exogenous cortisol on Ca^2+^ influx and Ca^2+^ transporter expression in tilapia larvae

**Fig. 5:**
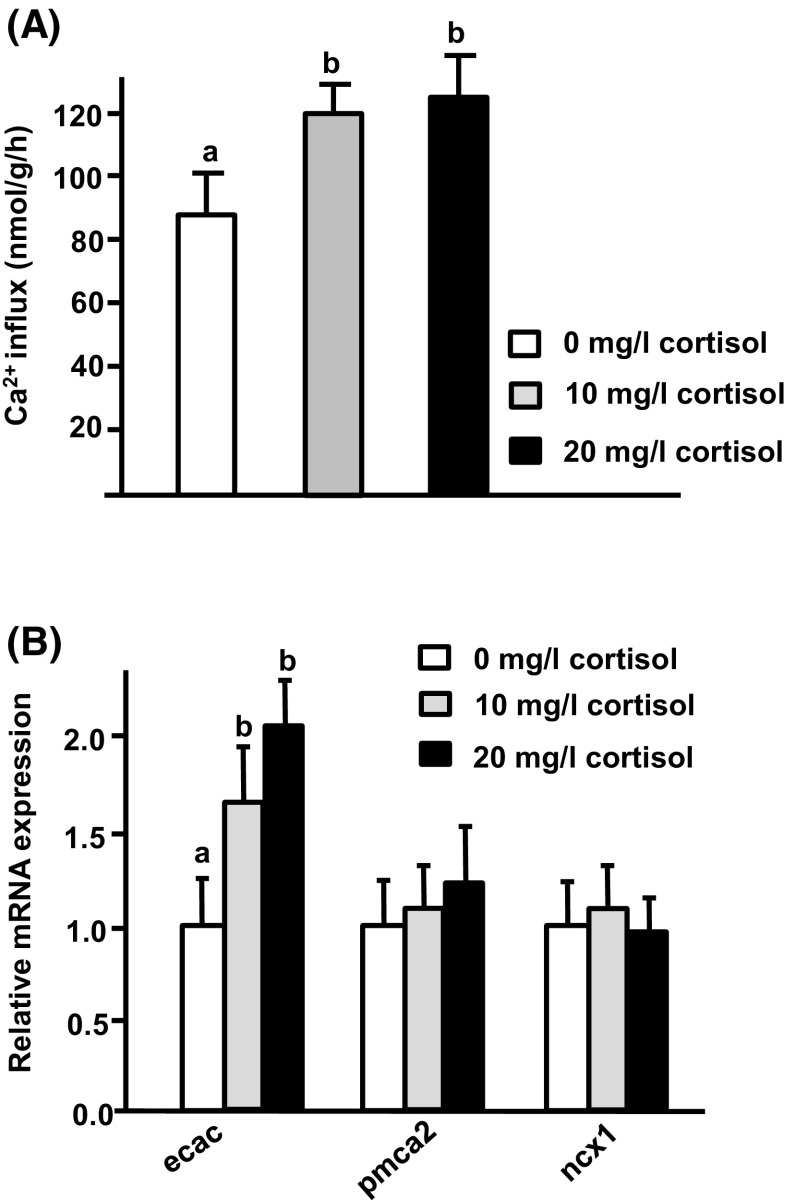
Effects of exogenous cortisol on Ca^2+^ influx and mRNA expression of transporters in tilapia larvae. Expression of mRNA was analyzed by qPCR, and values were normalized to β-actin. ^ab^Indicate a significant difference (*p* < 0.05) using Tukey’s multiple comparison test following one-way ANOVA. Values are the mean ± SD (*n* = 6)

**Fig. 6:**
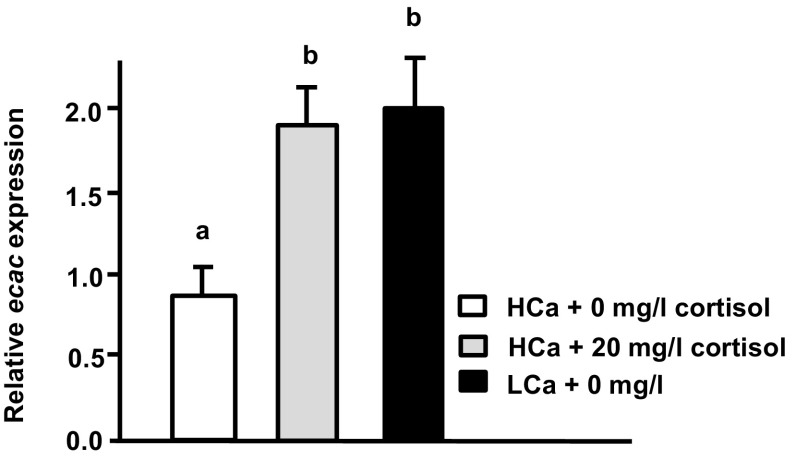
Effects of exogenous cortisol on *ecac* expression in tilapia larvae exposed to high Ca^2+^. Expression of mRNA was analyzed by qPCR, and values were normalized to β-actin. ^ab^Indicate a significant difference (*p* < 0.05) using Tukey’s multiple comparison test following one-way ANOVA. Values are the mean ± SD (*n* = 6)

As described above, expression of *cyp11b*, which encodes a cortisol synthesis enzyme, was enhanced in low Ca^2+^ water. The effect of cortisol on Ca^2+^ uptake of tilapia was further examined by treating hatching tilapia embryos with exogenous cortisol (0, 10, or 20 mg/l cortisol in local tap water) for 3 days. After the treatment period, larvae were sampled to analyze Ca^2+^ influx and transporter expression. We report that treatment with exogenous cortisol (10 and 20 mg/l) resulted in significant stimulation of Ca^2+^ influx and *ecac* expression, but did not modulate expression of *pmca2* or *ncx1b* (Fig. 5). Application of exogenous cortisol (20 mg/l cortisol in high Ca^2+^) also clearly enhanced *ecac* expression in tilapia larvae (Fig. 6). Furthermore, the enhanced level of *ecac* transcription was similar to that observed in tilapia larvae acclimated to low Ca^2+^ (Fig. 5b).

### Effects of GR or MR antagonist on *ecac* expression in tilapia larvae treated with exogenous cortisol

**Fig. 7:**
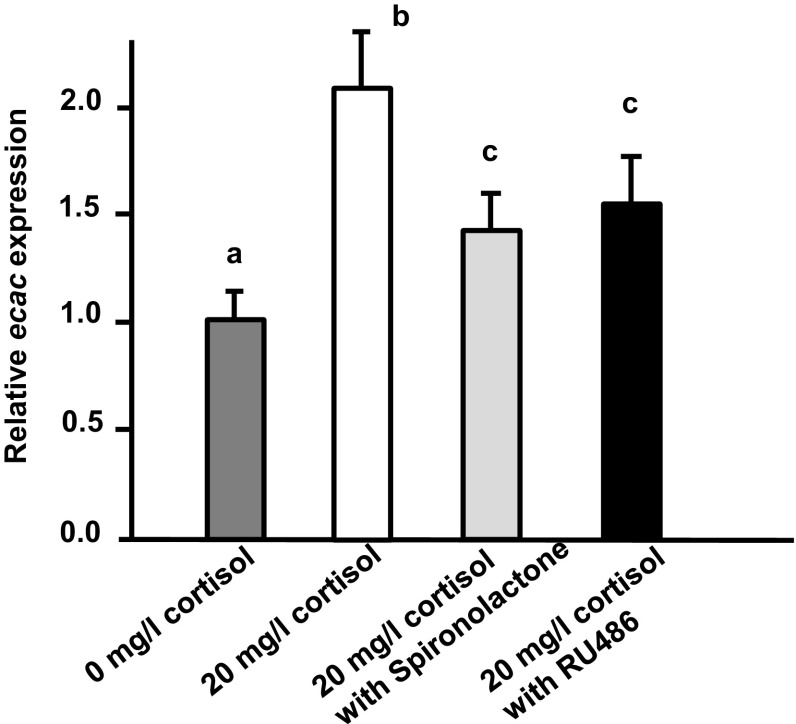
Effects of GR and MR antagonists on *ecac* expression in tilapia larvae treated with cortisol. Expression ofmRNA was analyzed by qPCR, and values were normalized to β-actin. ^abc^Indicate a significant difference (*p* < 0.05) using Tukey’s multiple comparison test following one-way ANOVA. Values are the mean ± SD (*n* = 6)

The regulatory mechanism of cortisol on Ca^2+^ uptake in tilapia was clarified by the exposing cortisol-treated tilapia larvae to 10 µg/ml RU486 or spironolactone (GR and MR receptor antagonists, respectively). We report that treatment with either GR or MR antagonist dramatically decreases the stimulatory effect of exogenous cortisol on *ecac* transcription (Fig. 7).

## Discussion

In the present study, expression of *ecac*, *pmca2*, and *ncx1* was detected in several tissues in tilapia (Fig. 1), consistent with observations in zebrafish (Pan et al. 2005; Liao et al. 2007). Universal expression of these Ca^2+^ transporters in tilapia may be related to the maintenance of intracellular Ca^2+^ homeostasis, similar to the reported situation in mammals (Lee et al. 1994; Guerini 1998). Ionocytes in the adult gills or the larval skin are vital sites for Ca^2+^ uptakein fish (Flik et al. 1995; Hwang et al. 2011). Liao et al. (2007) first identified mRNA signals of *ecac*, *pmca2*, and *ncx1b* in ionocytes of zebrafish, thereby providing comprehensive molecular evidence for the model of transcellular epithelial Ca^2+^ transport in ionocytes. The present study also identified *ecac*, *pmca2*, and *ncx1* mRNA signals in ionocytes of tilapia (Fig. 2), in agreement with the findings of Liao et al. (2007). In zebrafish, there are at least four subtypes of ionocytes, and they are specifically responsible for the regulation of (1) Cl^−^, (2) Na^+^, (3) Ca^2+^, and (4) K^+^ and acid–base, respectively (Hwang and Chou 2013). There are also four subtypes of ionocytes in tilapia (Inokuchi et al. 2009; Hwang et al. 2011). Herein, we observed that some ionocytes express ECaC mRNA (Fig. 2c): these *ecac*-expressing ionocytes may belong to a previously identified subtype or a new subtype. However, technical limitations prevented us from further classifying the *ecac*-expressing cells in the present study. This issue awaits further exploration in the future.

In the present study, normalized branchial *ecac* expression was observed to be higher (over ~300 fold at least) than that of *pmca2* and *ncx1* in tilapia (Fig. 3b). In zebrafish gills, the normalized mRNA expression of ECaC is also much higher than that of NCX1b and PMCA2 (Liao et al. 2007). In fact, several studies have shown that ECaC plays a dominant role in fish Ca^2+^ regulation. In zebrafish, a loss-of-function mutation of the ECaC gene resulted in a significant decrease of Ca^2+^ content and defective bone structure (Vanoevelen et al. 2011). Treatment with low Ca^2+^ water stimulated both Ca^2+^ absorption and *ecac* expressionin zebrafish, but did not affect the expression of NCX1b and PMCA2 (Pan et al. 2005; Lin et al. 2011, 2012, 2014; Lafont et al. 2011). Similarly, low Ca^2+^ medium treatment also stimulated branchial protein and mRNA expression of ECaC in trout; intra-arterial infusion with CaCl_2_ was found to suppress gill *ecac* expression in trout (Shahsavarani and Perry 2006). In the present study, normalized branchial *ecac* expression was observed to be higher than that of *pmca2* and *ncx1* in tilapia (Fig. 3). Moreover, low Ca^2+^ water treatment stimulated Ca^2+^ uptake and *ecac* expression in both adult and larval tilapia. These results further reinforced the findings of previous studies that indicate that modulation of *ecac* expression is vital for teleosts to cope with environmenta lCa^2+^ challenges.

Many studies have indicated that hormones and signal transduction pathways may be involved in the adjustment of Ca^2+^ uptake in fish upon Ca^2+^ challenge (Evans et al. 2005; Lin et al. 2012, 2014). Cortisol is a hypercalcemic hormone, but there is little molecular evidence for the effects of cortisol on fish Ca^2+^ uptake (Shahsavarani and Perry 2006; Lin et al. 2011). Here, expression of *cyp11b*, which encodes the enzyme required for the final step of cortisol synthesis, was found to be significantly upregulated by low Ca^2+^ medium treatment in tilapia (Fig. 4b). Exogenous cortisol treatment was found to cause upregulation of both Ca^2+^ influx and *ecac* expression in tilapia (Fig. 5). Moreover, exogenous cortisol treatment also enhanced *ecac* expression in tilapia larvae treated with high Ca^2+^ (Fig. 6). These results indicate that cortisol is a hypercalcemic hormone in tilapia, reinforcing the findings in other species. Lin et al. (2011) revealed that expression of both *cyp11b* and *ecac* is stimulated in zebrafish embryos treated with low Ca^2+^. Exogenous cortisol treatment was previously reported to enhance Ca^2+^ uptake through increasing *ecac* expression in zebrafish (Lin et al. 2011). In trout, exogenous cortisol treatment was also reported to stimulate branchial Ca^2+^ uptake or *ecac* expression (Flik and Perry 1989; Shahsavarani and Perry 2006; Kelly and Wood 2008). Taken all together, it appears that the hypercalcemic effects of cortisol are of physiological significance in terms of fish body fluid Ca^2+^ homeostasis.

Cortisol is the main corticosteroid hormone and may exert its actions through GR and/or MR in fish. Although several studies have addressed cortisol’s effects on Ca^2+^ regulation, only the earlier study by Lin et al. (2011) precisely investigated the role of cortisol receptor in Ca^2+^ regulation in the zebrafish model. Cortisol was demonstrated to increase Ca^2+^ uptake through GR alone, and protein and mRNA expression of GR was identified in Na^+^-K^+^-ATPase-rich cells (i.e., *ecac*-expressing ionocytes) in zebrafish (Lin et al. 2011; Cruz et al. 2013b). Prior to the current study, it was unknown whether GR (or MR) mediated the effects of cortisol on Ca^2+^ uptake in teleosts other than zebrafish. Here, we exposed cortisol-treated tilapia larvae with either GR or MR antagonists, both of which could antagonize the stimulatory effect of exogenous cortisol on *ecac* expression (Fig. 7); these findings imply that both GR and MR mediate the effect of cortisol on *ecac* expression in tilapia. A previous study reported that GR and MR mRNA are expressed in the branchial ionocytes of tilapia (Aruna et al. 2012). This result raises the possibility that cortisol may directly regulate *ecac* expression in tilapia. In the present study, spironolactone was used as a MR antagonist; however, the dispute about antagonist property of spironolactone existed in different fish species or experiment designs. Spironolactone revealed antagonist property in the gills of killifish with freshwater acclimation and cultured gills of salmon (Scott et al. 2005; Kiilerich et al. 2007), and it appears to also functionas an antagonist in the present study. In trout, spironolactone showed antagonist and agonist properties in vivo and in vitro studies, respectively (Sloman et al. 2001; Sturm et al. 2005). On the other hand, spironolactone acted as an agonist to the zebrafish MR overexpressed in mammalian cell lines (Pippal et al. 2011) and did not show effect on the gills of killifish with seawater acclimation (Shaw et al. 2007). To reinforce the present study’s findings, it is necessary to further clarify the property of spironolactone on tilapia MR in cell line experiments in the future.

For summary, the mRNA expression of three Ca^2+^ transporters (ECaC, PMCA2, and NCX1) was specifically detected in branchial ionocytes and exposure to low Ca^2+^ water resulted in significant stimulation of both Ca^2+^ influx and *ecac* expression in tilapia, similar to the previous findings in zebrafish (a stenohaline species) (Liao et al. 2007; Lin et al. 2011). One of the underlying mechanisms is probably the GR and/or MR-mediated hypercalcemic action of cortisol in tilapia (a euryhaline species), different from the GR-mediated mechanism in zebrafish as reported previously. From the point of view of comparative physiology, the present study enhances our understanding ofthe effects of cortisol on fish body fluid Ca^2+^ homeostasis.
